# Association of Sociodemographic Parameters With Depression, Anxiety, Stress, Sleep Quality, Psychological Trauma, Mental Well-Being, and Resilience During the Second Wave of COVID-19 Pandemic: A Cross-Sectional Survey From India

**DOI:** 10.7759/cureus.16420

**Published:** 2021-07-16

**Authors:** Tanveer Kaur, Piyush Ranjan, Avinash Chakrawarty, Keerthana Kasi, Parul Berry, Suryansh Suryansh, Archisman Mazumder, Munnoo Khan, Ashish D Upadhyay, Gaurishanker Kaloiya, Siddharth Sarkar, Vijay B Prasad

**Affiliations:** 1 Medicine, All India Institute of Medical Sciences, New Delhi, IND; 2 Geriatric Medicine, All India Institute of Medical Sciences, New Delhi, IND; 3 Psychology, Morarji Desai National Institute of Yoga, New Delhi, IND; 4 Statistics, All India Institute of Medical Sciences, New Delhi, IND; 5 Clinical Psychology, All India Institute of Medical Sciences, New Delhi, IND; 6 Addiction, All India Institute of Medical Sciences, New Delhi, IND; 7 Psychiatry/Clinical Psychology, All India Institute of Medical Sciences, New Delhi, IND

**Keywords:** mental health, psychology, well-being, dass-21, coronavirus, pandemic, psychological impact of a pandemic

## Abstract

Objective

This study was conducted to assess the traumatic impact of the second wave of coronavirus disease 2019 (COVID-19) pandemic on depression, anxiety, stress, sleep quality, mental well-being, and resilience among the general population of India.

Methods

An online cross-sectional survey was conducted in May-June, 2021 via Google Forms, which included adult individuals who were willing to participate in the study. The purposive and snowball sampling technique was used to ensure the principle of maximum diversity. Standardised tools [Depression Anxiety and Stress Scale (DASS), Pittsburgh Sleep Quality Index (PSQI), Impact of Event-Revised (IES-R), Short Warwick-Edinburgh Mental Well-being Scale (SWEMWBS), and the Brief Resilience Scale (BRS)] were used to collect data.

Results

A total of 1,109 responses were analysed for this study. Participants of different age groups (mean age: 32.98 ±14.72 years) and different sociodemographics were enrolled. The younger population group (18-34 years) was found to be the most affected among all the age groups. The findings revealed that 44.18% showed posttraumatic stress disorder (PTSD)-like symptoms. About 48.87%, 65.56%, and 22.09% of the participants had significant depression, anxiety, and stress symptoms respectively, and 11.27% had disturbed sleep patterns. Mental well-being was found to be disturbed for 74.75% of the study population, out of which only 4.15% showed high resilience capacity.

Conclusion

The associated collective psychological trauma mapped out by this paper is a pandemic in itself and needs to be addressed on a scale similar to the efforts being made to curb the physical symptoms of COVID-19.

## Introduction

The second and more intense wave of the coronavirus disease 2019 (COVID-19) pandemic has had a major impact on the mental health of individuals and communities [1,2]. The pervasiveness of the disease in this wave has meant that a majority of the individuals have been plagued by feelings of hopelessness and despair, irrespective of whether they have personally contracted the infection or not. For people who themselves fell sick or saw their loved ones battle COVID-19, caring for the affected in the family and often mourning the losses in their circles have led to an immense toll on psychological functioning across all age groups in the nation. Also, people who remained uninfected have had to bear witness to all this trauma and have become distressed about the consequences of COVID-19 [3,4].

The shortage of beds in hospitals, black marketing of medicines, lack of oxygen supply, and long queues outside the crematoriums have turned this wave into a collective trauma for many people [5]. The resultant inability to process the situation and feeling of helplessness has led to several psychological conditions such as depression, anxiety, stress, and disturbed sleep [6] and has been found to seriously impair the overall mental well-being of the individuals [7]. Since capacities for resilience among individuals vary and all of them cope differently with traumatic events, their paths back to normal functioning will also be diverse and varied [8].

In light of this, the objective of this study was to analyse the psychological impact of the second wave of the COVID-19 pandemic among the general population across India. The study aimed to arrive at a comprehensive assessment of the prevalence and diversity of psychological conditions in the wake of the newest wave of the pandemic so that further psychological screening and intervention can be designed by mental health professionals to eventually bring the nation back to normalcy.

## Materials and methods

Study design and rationale

This study was approved by the Institute Ethics Committee (IEC/689/6/2020), AIIMS, New Delhi. An online cross-sectional survey was conducted to assess the psychological impact of COVID-19 (second wave) among the general population across the nation. The survey was administered from May 24 to June 5, 2021, via Google Forms, for which the link was shared using various social media platforms such as WhatsApp, e-mail, Instagram, and Facebook. Telephonic interviews were conducted with those individuals who were unable to comprehend the questionnaires in English. Participants were informed about the objective of the study, which was also attached in the survey form as "Patient Information Sheet". A consent form was attached to the survey questionnaire that was circulated via Google Forms, and consent was obtained before enrollment in the study. The confidentiality of the responses was ensured and only completed questionnaires were considered for the statistical analysis. The principle of maximum diversity was followed to preserve maximum representation in the Indian sample. The purposive and snowball sampling technique was followed and various subject groups (based on age, gender, residence, and socioeconomic status) were determined using a quota sampling technique.

Participants

A total of 1,109 participants were analysed. Participants of various sociodemographic parameters such as age, gender, socioeconomic strata, residence, psychiatric comorbidities, and COVID-19 infection status were recruited for this study. The inclusion was limited to adult individuals who were interested and willing to participate in the study. Participants who were less than 18 years of age and those who did not consent were excluded from the study.

Questionnaires

The online survey was designed to assess the psychological impact of the COVID-19 pandemic among the general population across India. The survey was divided into six sections to assess the following variables: sociodemographic details; depression, anxiety, and stress; resilience; impact of event; sleep quality; and well-being.

Sociodemographics

The general information of the participants, such as name, age, gender, residence, socioeconomic status, marital status, and COVID-19 infection status were recorded in this section.

Depression Anxiety and Stress Scale-21 (DASS-21)

The DASS-21 consists of three sub-scales to measure negative emotional states (depression, anxiety, and stress). Each subscale consists of seven items. The scale has excellent Cronbach's alpha values of 0.81, 0.89, and 0.78 for the subscales of depression, anxiety, and stress respectively along with internal consistency and concurrent and convergent validities [9].

Pittsburgh Sleep Quality Index (PSQI)

The PSQI is a self-reported instrument that measures the quality of sleep as well as sleep disturbances over the period of one month. The scale assesses seven domains: sleep quality, sleep duration, sleep latency, habitual sleep efficiency, sleep disturbances, use of sleep medication, and daytime dysfunction. The Cronbach’s alpha for the seven domains is 0.83, indicating high internal consistency [10].

Impact of Event-Revised (IES-R)

The IES-R is a self-administering tool containing 22 items. It helps to assess acute stress, everyday trauma, and routine life stress during the pandemic. The scale consists of three subscales: intrusion (eight items), avoidance (eight items), and hyperarousal (six items), which show a high degree of intercorrelation (r=.52 to .87), and the test-retest reliability is found to be 0.89-0.94 over a period of six months [11].

Short Warwick-Edinburgh Mental Well-Being Scale (SWEMWBS)

The SWEMWBS is used to measure psychological functioning and emotional well-being. It is a shortened version of WEMWBS, comprising seven items. The scale has a good psychometric property coupled with internal consistency (α=0.89) [12].

Brief Resilience Scale (BRS)

The ability to bounce back or recover from stress is measured using this tool. The scale consists of six items rated on a five-point Likert scale. The scale has good internal consistency with Cronbach’s alpha ranging between 0.80-0.91 and test-retest reliability between 0.62-0.69 [13].

Data and statistical analysis

Descriptive statistics such as frequency and percentages were calculated for sociodemographics. The mean and standard deviations were calculated for the continuous variables. Association between sociodemographics and study variables was calculated using the chi-square test. The analyses were performed using STATA/SE version 14.2 (StataCorp LP, College Station, TX).

## Results

Sociodemographic profile of participants

We received a total of 1,115 responses, out of which six were duplicate entries and were eliminated before the statistical analysis. The sociodemographic profiles of 1,109 participants who were ultimately included are depicted in Table 1.

**Table 1:** General characteristics of the study participants COVID-19: coronavirus disease 2019

Characteristics	Variables, n (%)
Age group (years)	
18-34	685 (61.77)
35-59	348 (31.38)
60+	76 (6.85)
Gender	
Male	525 (47.34)
Female	584 (52.66)
Area of residence	
Metropolitan	388 (34.99)
City	529 (47.70)
Village	192 (17.31)
Whether healthcare worker	
Yes	299 (26.96)
No	810 (73.04)
Marital status	
Married	527 (47.52)
Unmarried	582 (52.48)
Family type	
Joint	352 (31.74)
Nuclear	757 (68.26)
Socioeconomic status	
Low	282 (25.43)
Middle	373 (33.63)
High	454 (40.94)
Diagnosed case of psychiatric illness	
Yes	51 (4.60)
No	1,058 (95.40)
COVID-19 infection status	
Self infected	112 (10.10)
Family infected	203 (18.30)
Self and family infected	136 (12.26)
None	658 (59.34)
Death of close friends or relatives due to COVID-19	
Yes	514 (46.35)
No	595 (53.65)
Whether the infected friend or family member was able to get COVID-19 treatment	
Yes	450 (40.58)
No	202 (18.21)
Not applicable	457 (41.21)

The mean age of the participants was found to be 32.98 ±14.72 years. There was a fair representation of both the genders as well as married and unmarried participants. There was a predominance of participants of younger age (18-34 years) and those belonging to higher socioeconomic strata and residing in the urban areas. Only one-third of the population were healthcare workers (frontline workers, paramedics, etc.), and only 4.60% had clinically diagnosed psychiatric illnesses. Approximately half of the participants reported that they had lost a close family member or friend to COVID-19.

Depression, anxiety, and stress

Depression

The results of the DASS-21 revealed that approximately half (48.87%) of the study population was clinically depressed. A significant association was found between depression and various sociodemographic factors as depicted in Table 2. More than half of the total participants (54.74%) who had mild to extremely severe depression were in the age group of 18-34 years (χ2=33.57; p<0.001). Unmarried participants (χ2=36.84; p<0.001) and those belonging to urban areas (χ2=27.31; p<0.001) and higher sections of the society (χ2=28.21; p<0.001) were found to be more in the clinical domain of depression in comparison to the others. Approximately half of the participants (47.53%) who were not diagnosed with any prior psychiatric illness were found to have depression of varying intensities (χ2=29.36; p<0.001).

Anxiety

The findings showed that more than half of the participants (65.56%) had symptoms of anxiety ranging from mild to extremely severe. A significant association was established between anxiety and various sociodemographics, as shown in Table 2. The younger population group (18-34 years) was found to be more anxious in comparison to other age groups (χ2=20.77; p<0.01). The unmarried (χ2=13.87; p<0.01), those belonging to the higher section of the society (χ2=38.54; p<0.001), and those residing in the metropolitan and urban areas (χ2=23.87; p<0.01) showed higher symptoms of anxiety. Study subjects who had lost their close ones in the pandemic were found to be more anxious in comparison to the others (χ2=46.34; p<0.001).

Stress

The results revealed that only one-fifth (22.09%) of the participants had symptoms of significant stress. Table 2 shows the association between stress and different sociodemographics. Similar to the previously evaluated domains (depression and anxiety), the younger population group was found to be more stressed with symptoms ranging from mild to severe (χ2=20.49; p<0.01). Participants who did not have any clinically diagnosed psychiatric illness were found to have mild stress (χ2=27.31; p<0.001). Those who witnessed the death of a friend or a relative due to the COVID-19 infection (χ2=16.64; p<0.001) and those who could not arrange treatment for their infected family members (χ2=22.96; p<0.001) were found to be significantly stressed.

Sleep Quality

The quality of sleep was found to be disturbed for only 11.27% of the population. A significant association between sleep quality and various sociodemographics was observed, which is well depicted in Table 2. There was a noteworthy deterioration in sleep quality for the younger population group (18-34 years) followed by the age group of 35-59 years, in comparison to the elderly population. Married participants (χ2=7.70; p<0.01) and those belonging to low socioeconomic status (χ2=27.88; p<0.001) were found to have disturbed sleep more frequently.

Impact of Event [posttraumatic stress disorder (PTSD)]

The scores of IES-R revealed that 44.18% of the total population showed PTSD-like symptoms and one-fourth (25.25%) of the study participants showed severe PTSD symptoms. The younger population group (18-34 years) was more likely to have PTSD-like features (χ2=14.57; p<0.05). The COVID-19 pandemic was more likely to have a traumatic impact on those residing in metropolitan and urban areas (χ2=21.28; p<0.01) in comparison to the villagers and those belonging to high socioeconomic status (χ2=19.70; p<0.01) in comparison to low socioeconomic status group. Participants who had lost their close ones in this pandemic were found to be disturbed with symptoms ranging from mild to severe intensities (χ2=35.37; p<0.001).

Well-Being

Two-thirds (74.75%) of the study population reported a low sense of well-being, out of which 29.49% and 7.84% reported possible and probable depression respectively on SWEMWBS. The association of well-being with various sociodemographics can be seen in Table 2. The young adults in the age group of 18-34 years (χ2=53.84; p<0.001), the unmarried (χ2=40.50; p<0.01), and those belonging to low socioeconomic status (χ2=57.71; p<0.01) reported a significantly lower sense of well-being. The death of a close family member or friend pushed the majority of the participants (51.07%) into the category of possible depression.

Resilience

The results of the BRS revealed that a very small proportion of the study participants (4.15%) had high resilience. It was seen that the young population (18-34 years) was found to have low resilience (χ2=24.72; p<0.001) as compared to the older population. Females (59.65%) and unmarried participants (64.91%) were found to have low resilience in comparison to males and married participants respectively (χ2=15.18; p<0.001 and χ2=24.08; p<0.001). The findings also indicated that the general population in comparison to healthcare workers had low resilience with respect to the whole situation of the second wave of COVID-19 pandemic (χ2=6.21; p<0.05). The association between resilience and sociodemographics can be seen in Table 2.

**Table 2:** Association of psychological domains with sociodemographics COVID-19: coronavirus disease 2019; NS: not significant; PTSD: posttraumatic stress disorder; SES: socioeconomic status

Domain	Frequency of responses by participants (%)	Association with sociodemographic correlates									
												Age	Gender	Marital status	Residence	SES	Family type	Healthcare worker	Psychiatric comorbidity	Death due to COVID-19	Availability of treatment
		Depression		P<0.001	NS	P<0.001	P<0.001	P<0.001	NS	P<0.05	P<0.001											P<0.001	P<0.001
		Normal	567 (51.13)																				
Mild	255 (22.99)																						
Moderate	207 (18.67)																						
Severe	74 (6.67)																						
Extremely severe	6 (0.54)																						
Anxiety		P<0.01	NS	P<0.01	P<0.01	P<0.001	NS	NS	P<0.001	P<0.001	P<0.001
Normal	382 (34.45)										
Mild	221 (19.93)										
Moderate	352 (31.74)										
Severe	108 (9.74)										
Extremely severe	46 (4.15)										
Stress		P<0.01	NS	P<0.001	P<0.01	P<0.01	NS	NS	P<0.001	P<0.01	P<0.001
Normal	864 (77.91)										
Mild	142 (12.8)										
Moderate	90 (8.12)										
Severe	13 (1.17)										
Extremely severe	0 (0)										
Impact of event (PTSD)		P<0.05	NS	NS	P<0.01	P<0.01	NS	NS	P<0.001	P<0.001	P<0.001
Minimal	619 (55.82)										
Mild	159 (14.34)										
Moderate	51 (4.6)										
Severe	280 (25.25)										
Well-being		P<0.001	NS	P<0.001	NS	P<0.001	P<0.05	P<0.05	NS	P<0.001	P<0.001
High	280 (25.25)										
Average	415 (37.42)										
Possible depression	327 (29.49)										
Probable depression	87 (7.84)										
Sleep quality		P<0.01	NS	P<0.01	NS	P<0.001	NS	NS	NS	P<0.001	NS
Normal	984 (88.73)										
Disturbed	125 (11.27)										
Resilience		P<0.001	P<0.001	P<0.001	NS	NS	NS	P<0.05	P<0.05	NS	NS
Low	285 (25.7)										
Medium	778 (70.15)										
High	46 (4.15)										

Status of COVID-19 Infection and Psychological Domains

Our study revealed significant associations between the status of COVID-19 infection and five psychological domains (Table 3). More than half of the study population who were either infected themselves (58.04%) or had any of the family member(s) infected (60.59%) or both (self and family members infected) (61.76%) were found to be clinically depressed in varying intensities (χ2=62.10; p<0.001). Similarly, a majority of the patients who had COVID-19 infection themselves (78.57%) or had family member(s) with COVID-19 (75.37%) or both (77.94%) were found to have clinically significant levels of anxiety (χ2=68.62; p<0.001). A significant amount of the study population who had either family member(s) infected (34.48%) or had an infection in both self and family (36.03%) revealed a severe traumatic impact (χ2=50.15; p<0.001). Approximately three-fourths of the population experienced a negative impact on their well-being irrespective of their COVID-19 infection status (χ2=24.00; p<0.01).

**Table 3:** Association of COVID-19 infection status with psychological domains COVID-19: coronavirus disease 2019; NS: not significant; PTSD: posttraumatic stress disorder

Domain	Frequency of responses by participants (%) and association							
	No infection (n=658)	Only self infection (n=112)	Only family infection (n=203)	Infection in self and family (n=136)	Association	Infected in the first wave (n=140)	Infected in the second wave (n=254)	Association
									Depression								
									Normal	388 (58.97)	47 (41.96)	80 (39.41)	52 (38.24)	P<0.001	62 (44.29)	100 (39.37)	NS
Mild	141 (21.43)	28 (25.00)	51 (25.12)	35 (25.74)	37 (26.43)	61 (24.02)											
Moderate	92 (13.98)	32 (28.57)	44 (21.67)	39 (28.68)	29 (20.71)	65 (25.59)											
Severe	33 (5.02)	04 (3.57)	27 (13.30)	10 (7.35)	11 (7.86)	27 (10.63)											
Extremely severe	04 (0.61)	01 (0.89)	01 (0.49)	0 (0.00)	01 (0.71)	01 (0.39)											
Anxiety								
Normal	278 (42.25)	24 (21.43)	50 (24.63)	30 (22.06)	P<0.001	28 (20.00)	62 (24.41)	NS
Mild	140 (21.28)	21 (18.75)	40 (19.70)	20 (14.71)		31 (22.14)	45 (17.72)	
Moderate	175 (26.60)	48 (42.86)	76 (37.44)	53 (38.97)		60 (42.86)	95 (37.40)	
Severe	43 (6.53)	14 (12.50)	26 (12.81)	25 (18.38)		17 (12.14)	34 (13.39)	
Extremely severe	22 (3.34)	05 (4.46)	11 (5.42)	08 (5.88)		04 (2.86)	18 (7.09)	
Stress								
Normal	545 (82.83)	84 (75.00)	139 (68.47)	96 (70.59)	P<0.001	101 (72.14)	177 (69.69)	NS
Mild	68 (10.33)	20 (17.86)	29 (14.29)	25 (18.38)		24 (17.14)	39 (15.35)	
Moderate	38 (5.78)	08 (7.14)	29 (14.29)	15 (11.03)		12 (8.57)	35 (13.78)	
Severe	07 (1.06)	0 (0.00)	06 (2.96)	0 (0.00)		03 (2.14)	03 (1.18)	
Extremely severe	0 (0.00)	0 (0.00)	0 (0.00)	0 (0.00)		0 (0.00)	0 (0.00)	
Impact of event (PTSD)								
Normal	414 (62.92)	66 (58.93)	87 (42.86)	52 (38.24)	P<0.001	70 (50.00)	112 (44.09)	NS
Mild	84 (12.77)	15 (13.39)	31 (15.27)	29 (21.32)		22 (15.71)	42 (16.54)	
Moderate	27 (4.10)	03 (2.68)	15 (7.39)	06 (4.41)		08 (5.71)	15 (5.91)	
Severe	133 (20.21)	28 (25.00)	70 (34.48)	49 (36.03)		40 (28.57)	85 (33.46)	
Well-being								
High	160 (24.32)	29 (25.89)	51 (25.12)	40 (29.41)	P<0.01	34 (24.29)	75 (29.53)	NS
Average	243 (36.93)	42 (37.50)	77 (37.93)	53 (38.97)		56 (40.00)	87 (34.25)	
Possible depression	185 (28.12)	32 (28.57)	67 (33.00)	43 (31.62)		42 (30.00)	84 (33.07)	
Probable depression	70 (10.64)	09 (8.04)	08 (3.94)	0 (0.00)		08 (5.71)	08 (3.15)	
Sleep quality								
Normal	570 (86.63)	102 (91.07)	183 (90.15)	129 (94.85)	P<0.05	130 (92.86)	230 (90.55)	NS
Disturbed	88 (13.37)	10 (8.93)	20 (9.85)	07 (5.15)		10 (7.14)	24 (9.45)	
Resilience								
Low	160 (24.32)	26 (23.21)	58 (28.57)	41 (30.15)	NS	41 (29.29)	65 (25.59)	NS
Medium	474 (72.04)	78 (69.64)	137 (67.49)	89 (65.44)		95 (67.86)	174 (68.50)	
High	24 (3.65)	08 (7.14)	08 (3.94)	06 (4.41)		04 (2.86)	15 (5.91)	

## Discussion

The second wave of the COVID-19 pandemic has triggered serious health emergencies. These health emergencies are expected to affect the physical and the psychological well-being of both individuals as well as communities, which may translate into emotional reactions (depression, anxiety, stress, and other psychiatric conditions) [14-18]. Though several studies were conducted during the first wave to assess the psychological impact of COVID-19, our study aimed to assess the impact of the second wave.

We assessed the traumatic impact of the second wave of the pandemic using IES-R, and the results indicated that almost half of the participants (44.18%) experienced mild to severe impact of the pandemic, and 25.25% of the population reported severe impact. A study conducted during the first wave reported a comparatively lesser impact (33.2%) on the psychological health of the individuals (4). Another study reported that 28.2% of the participants showed PTSD-like symptoms [19].

This traumatic impact of the pandemic has led to people experiencing heightened emotional responses such as stress, depression, and anxiety. Our findings revealed that approximately half of the study population (48.87%) was clinically depressed, varying from mild to extremely severe intensities. Two-thirds of the participants were found to be anxious, whereas 31.74% had moderate symptoms of anxiety, while only 22.09% reported a significant level of stress. Our results are not consistent with the findings of the previously conducted studies, which reported higher stress (74.1%) and lesser intensity of anxiety (38.2%) and depression (10.5%) [7]. The findings imply that the second wave has had a more severe impact on the psychological health of the individuals, with approximately half of the participants meeting the criteria for clinical depression and anxiety.

Apart from these disturbances in the mood, a few people have also experienced changes in their quality of sleep (11.27%). The changes in sleep quality are associated with depressive symptoms and mood disturbances. The studies conducted during the first wave also showed deterioration in the quality of sleep (10%), which is consistent with the findings of our study [20]. Several studies have also been conducted in the past year to assess these changes in sleep patterns due to the COVID-19 pandemic [21].

These disruptions in emotional states, as well as sleeping patterns, affect the overall mental well-being of the individuals. The scores on SWEMWBS revealed that three-fourths of the population (74.75%) experienced a decline in their well-being with features ranging from an average sense of well-being to probable depression. A study conducted during the month of July 2020 revealed that approximately 41.6% of the study participants had disturbed mental well-being [22]. This deterioration in mental well-being makes the population vulnerable to several psychiatric conditions in the long run [23].

In order to deal with these psychological and emotional changes due to the pandemic, it is important for the individuals to have high resilience, i.e., the capacity to bounce back to their normal functioning. The scores on the BRS showed that 74.3% of our population had medium to high resilience capacity, where 70.15% possess medium resilience capacity. Behavioural and psychological interventions such as lifestyle modification to improve sleep quality, as well as physical exercise, can significantly aid individuals in returning to their normal or pre-COVID-19 routine and mental states [24-28].

The major highlights from the study revealed that the younger population group, those residing in the urban areas and belonging to high socioeconomic status are found to be more vulnerable to mental health issues. To leverage India’s demographic dividend, it is essential to urgently address the emotional recuperation of its youth and working-class urban population in particular, who have been the worst affected by the pandemic.

Limitations and strengths

Since the survey was circulated through social media platforms and required access to smartphones and the internet to participate, it could reach the lower sections of society in only a limited way, which hampered its representativeness to some extent. Secondly, since the data was collected through purposive and snowball sampling techniques, the results cannot be generalised to the entire population. Despite these limitations, this is among the first studies to assess the psychological impact of the second wave of COVID-19 in India. We used standardised tools for the purpose of data collection, and these findings can be used to develop interventions as well as for planning any longitudinal study in the future.

## Conclusions

This study was conducted to assess the psychological impact of the second wave of the COVID-19 pandemic. The findings can be used by health policymakers and mental health professionals to frame a systemic response to the new emotional needs generated by the pandemic. The unprecedented collective psychological trauma mapped out by this paper is a pandemic in itself and needs to be addressed on a scale similar to the efforts being made to curb the physical symptoms of the virus.
